# A Novel Technique for Corneal Transepithelial Electrical Resistance Measurement in Mice

**DOI:** 10.3390/life14081046

**Published:** 2024-08-22

**Authors:** Yasser Helmy Mohamed, Masafumi Uematsu, Mao Kusano, Daisuke Inoue, Diya Tang, Keiji Suzuki, Takashi Kitaoka

**Affiliations:** 1 Department of Ophthalmology and Visual Sciences, Graduate School of Biomedical Sciences, Nagasaki University, Nagasaki 852-8501, Japan; yasserhelmy@nagasaki-u.ac.jp (Y.H.M.); maok@nagasaki-u.ac.jp (M.K.); d.i.private.3@gmail.com (D.I.); bb55319802@ms.nagasaki-u.ac.jp (D.T.); tkitaoka@nagasaki-u.ac.jp (T.K.); 2 Department of Radiation Medical Sciences, Atomic Bomb Disease Institute, Nagasaki University, Nagasaki 852-8523, Japan; kzsuzuki@nagasaki-u.ac.jp

**Keywords:** epithelial tight junctions, mice, transepithelial electrical resistance, ZO-1

## Abstract

We developed a technique that can measure corneal transepithelial electrical resistance (TER) in mice, which was used for evaluating corneal toxicity induced by ophthalmic drugs. We used a tissue culture well and its insert to mount the mouse globe and separated the cornea from the rest of the globe to enable corneal TER measurements to be taken. The explanted mouse eyes were divided into groups, and the corneal epithelia were exposed to different concentrations of BAC. Half of these eyes were fixed for transmission electron microscopy (TEM) examination and the other for ZO-1 immunohistochemical (IHC) evaluation. After exposure to control, 0.1%, 0.2%, and 0.5% BAC, the TER was 100 ± 0%, 91 ± 14%, 83 ± 13%, and 34 ± 12% of the pre-exposure TER at 1 min, respectively, with a statistically significant decrease in the 0.5% group. After 3 min, the TER showed a statistically significant decrease in the 0.2% and 0.5% groups. The TEM examinations showed a loss of epithelial tight junctions between superficial cells in the 0.2% and 0.5% groups. The IHC examination showed decreased ZO-1 staining of the corneal epithelium of the same groups as compared to the control. To the best of our knowledge, we succeeded in developing an innovative technique for corneal TER measurement in mice.

## 1. Introduction

The corneal epithelium, located on the outer surface of the cornea, serves as a protective barrier against external environmental factors. Nutrients are delivered through diffusion across both the epithelial and endothelial layers, maintaining proper homeostasis [1]. The tight junctions within the corneal epithelium, located at the most apical intercellular level, are crucial for establishing and maintaining the barrier function [2,3]. When the corneal epithelial barrier is compromised, it can lead to ocular irritation [4,5] and an elevated risk of microbial infections [6].

The electrophysiological properties of a cell or tissue can be assessed by passing an electric current through the tissue and measuring the resulting voltage drop and potential difference. When the delivered current and the measured voltage are known, the tissue’s resistance can be calculated using Ohm’s Law: resistance (R) is equal to the voltage (V) divided by the current (I in amperes) [7].

Corneal transepithelial electric resistance (TER) is maintained by corneal superficial cells with tight junctions between them, acting as a robust barrier against external invasion. Monitoring changes in corneal TER provides a quantitative assessment of the epithelial state and its barrier function. This method is particularly useful for evaluating the corneal toxicity induced by ophthalmic drugs. By continuously measuring corneal TER, we can quantitatively assess corneal permeability and irritancy [8,9].

Previously, we designed an in vivo corneal TER measurement system using custom-designed thin stick electrodes and a volt–ohm meter, which allowed us to assess the barrier function of the intact cornea in rabbits more accurately. By mimicking the clinical instillation of ophthalmic drugs, we obtained relevant data on the acute corneal toxicity associated with certain eye drops [10,11,12]. To address the clinical limitations associated with intraocular electrodes, we improved the corneal TER measurement method by developing electrodes that could be placed directly on the cornea and in the conjunctival sac [13]. We also developed a novel, less-invasive method for measuring corneal TER in humans. This approach allows us to directly assess the effect of various eye drops on the corneal epithelium in a safe and reliable manner [14].

While rabbits have traditionally been the preferred animal model for ophthalmic research, mice have gained popularity in recent years in the study of corneal pathologies and those of other ocular regions like the retina and crystalline lens [15,16]. The mouse’s widespread adoption is attributed to its fully sequenced genome and the availability of various transgenic and knockout strains for diverse research purposes. Moreover, mice are easy to breed, exhibit rapid growth, and offer cost-effective experimental options [17,18]. To the best of our knowledge, this is the first study to innovate TER measurements in the cornea of mice.

In rabbits, we used an intraocular electrode to measure TER, which is nearly impossible with mice because of the small globe. In this study, we developed an innovative technique to enable corneal TER measurements in mice. In addition, we tried to evaluate acute corneal permeability change after the exposure of mouse corneas to different concentrations of benzalkonium chloride (BAC) using our newly developed technique.

## 2. Materials and Methods

### 2.1. Chemicals and Antibodies

Ca^2+^- and Mg^2+^-free Hank’s Balanced Salt Solution (HBSS) was obtained from Invitrogen Corp. (Carlsbad, CA, USA). BAC 10% solution (mixed BAC) was obtained from Wako Pure Chemical Co. (Osaka, Japan). Mouse monoclonal antibodies to ZO-1 were obtained from EMD Millipore Corporation (Merck KGaA, Darmstadt, Germany), and Alexa Fluor 488-labeled donkey antibodies to mouse immunoglobulin G were obtained from Invitrogen (Thermo Fischer Scientific, Odgen, UT, USA).

### 2.2. Experimental Animals

Male ICR mice were kept in cages at the Laboratory Animal Center for Biomedical Research, Nagasaki University School of Medicine, under controlled conditions of temperature (21 °C), humidity (50 ± 5%), and a 12:12 h light/dark cycle. The mice were kept individually in cages free of wood debris for one week to prevent epithelial damage before the experiment. The mice were treated in compliance with the ARVO Statement for the Use of Animals in Ophthalmic and Vision Research.

### 2.3. TER Measurement Technique

**Figure 1:**
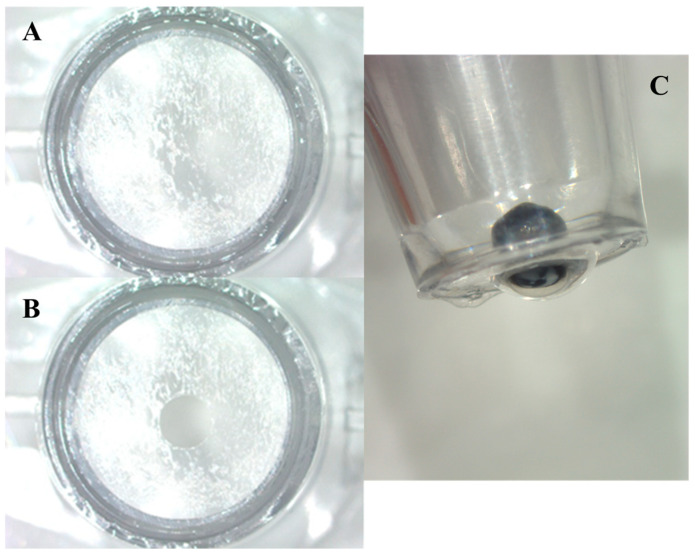
Cell culture insert bottom covered with biomedical adhesive (**A**). Cell culture insert bottom with 2.5 mm hole after punch use (**B**). Mouse cornea protruding from the hole in the cell culture bottom (**C**).

**Figure 2:**
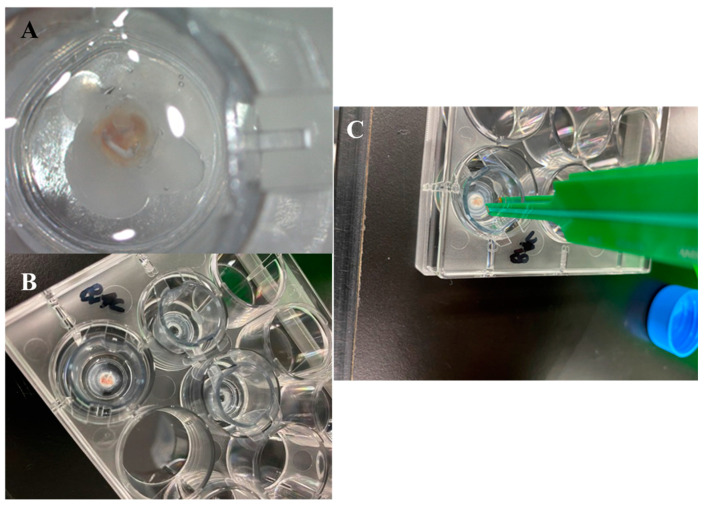
HBSS was poured into the cell culture insert above the eyeball after its isolation with Vaseline (**A**). The cell insert inside one cavity of a 12-well plate containing either HBSS or BAC (**B**). One of the two electrodes is inserted into the cell culture insert and the other in the 12-well plate (**C**).

**Figure 3:**
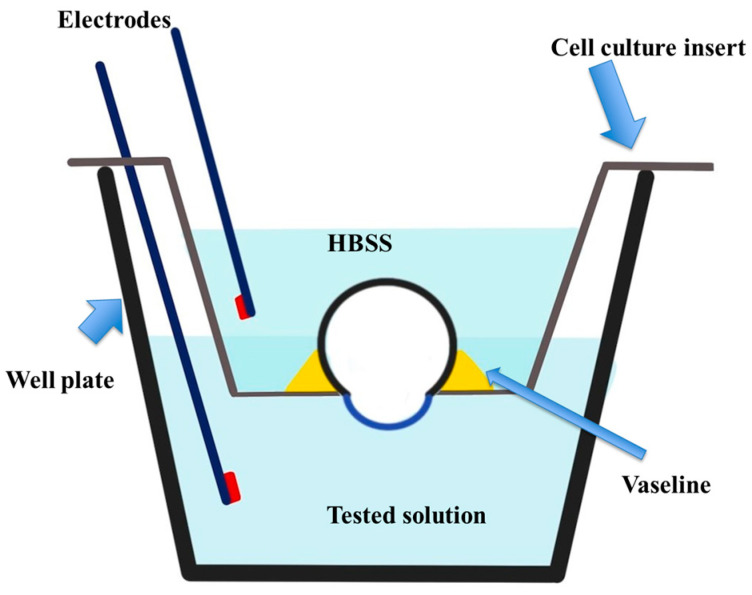
This schematic figure illustrates the TER measurements in mice. HBSS was poured into the cell culture insert above the eyeball after its isolation with Vaseline. The cell insert was placed inside one cavity of a 12-well plate containing either HBSS (control) or BAC (tested solution). One of the two electrodes was inserted into the cell culture insert and the other into the 12-well plate.

The mice were euthanatized, and the whole eyes were carefully enucleated and immersed in sterile HBSS. We applied and spread biomedical adhesive (Alon-Alpha A, Sankyo, Tokyo, Japan) on the cell culture insert bottom to ensure the closure of its pores (Figure 1A). We used a 2.5 mm biopsy punch to punch a hole in the central part of the cell culture insert bottom (Figure 1B). We used fine-toothed forceps to hold the eye from its optic nerve and mount the eye inside the hole in the cavity of the cell culture insert so that the mouse cornea protruded from the hole (Figure 1C). Melted Vaseline was poured carefully around the eye from inside the insert to isolate the cornea and was left to solidify. Then, 2 mL of HBSS was poured into the cell culture insert above the eyeball (Figure 2A). The cell insert was placed into one cavity of a 12-well plate containing either HBSS (control group) or BAC (test group) (Figure 2B). One of the two electrodes (Chopstick Electrode, World Precision Instruments, Sarasota, FL, USA) was inserted into the cell culture insert and the other into the 12-well plate (Figure 2C). Figure 3 illustrates how we carried out the TER measurements in the mouse corneas. The TER was monitored in real time using an EVOMX volt–ohm meter (World Precision Instruments, Sarasota, FL, USA). The meter generated a ±20 μA AC square-wave current at 12.5 Hz. After measuring the TER of the cornea both before and after exposure, the results were expressed as a percentage relative to the pre-exposure TER value (100%).

In the present study, the explanted mouse eyes were divided into 4 groups, and the corneal epithelium of these groups were exposed to different concentrations of BAC (0.1%, 0.2%, and 0.5%) in addition to the control group (HBSS group). The corneal TER was measured after 1 and 3 min. The sample size for the corneal TER study was set at 4, which we found to be sufficient for statistical analyses in our previous TER studies [13,14].

### 2.4. Transmission Electron Microscopy (TEM) Examination

Half of the eyes (all groups after TER measurements) were fixed in glutaraldehyde, and the corneas were fixed (after dissection) with 4% glutaraldehyde in 0.05 M cacodylate buffer and processed for examination with a transmission electron microscope (Hitachi H300, Hitachi, Ibaragi, Japan).

### 2.5. ZO-1 Immunohistochemical Staining

The remaining half of the eye specimens were fixed in 4% paraformaldehyde and embedded in O.C.T. medium (Tissue Tek, Miles, Naperville, IL, USA). These samples were then frozen for cryosectioning. The resulting cryosections, each 6 mm thick, were cut perpendicular to the corneal epithelial axis.

To visualize specific proteins, primary antibodies (specifically, mouse monoclonal antibodies to ZO-1) were applied in a blocked buffer and incubated for 2 h at room temperature. After four washes in phosphate-buffered saline (PBS), the sections were incubated with a secondary antibody (Alexa Fluor 488-conjugated donkey antibodies to mouse IgG) at a 1:500 dilution in PBS for 1 h at room temperature.

Finally, the samples were thoroughly washed in PBS and mounted using a solution containing 10% glycerol and 1 µg/mL diamidino-2-phenylindole (DAPI). The resulting images were captured using fluorescent microscopy (DM-6000B, Leica, Mannheim, Germany) with a digital camera (DFC-350 FX, Leica, Mannheim, Germany) attached to the microscope.

### 2.6. Statistical Analysis

All results were expressed as the mean ± standard error of the experiments. Statistical comparisons were performed using the Tukey test for the TER measurements (*n* = 4). Values of *p* < 0.05 were considered to indicate statistical significance.

## 3. Results

### 3.1. Corneal TER

**Figure 4:**
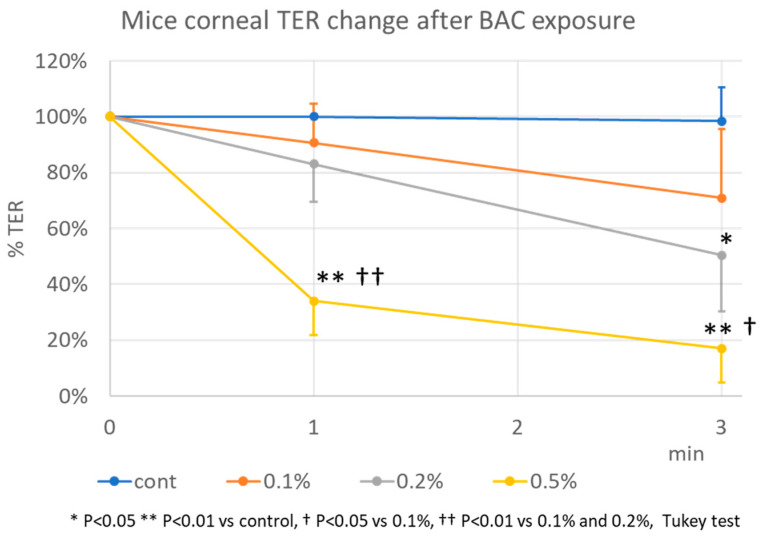
TER after mouse corneal exposure to HBSS and different concentrations of BAC solutions over time (* *p* < 0.05, ** *p* < 0.01 vs. control, † *p* < 0.05 vs. 0.1%, †† *p* < 0.01 vs. 0.1% and 0.2%, Tukey test).

The mean corneal TER for the mice used in this study was 482 ± 294 Ω cm^2^. Figure 4 shows the TER after corneal exposure to HBSS and different concentrations of BAC solutions. After exposure to control, 0.1%, 0.2%, and 0.5% BAC (*n* = 4), the TER was 100 ± 0%, 91 ± 14%, 83 ± 13%, and 34 ± 12% of the pre-exposure TER at 1 min, respectively, with a statistically significant decrease (*p* < 0.01) observed for the 0.5% BAC group. There was statistically significant decrease (*p* < 0.01) in the 0.1% and 0.2% BAC groups compared with the 0.5% BAC group. After 3 min, the TER was 98 ± 12%, 71 ± 25%, 50 ± 20%, and 17 ± 12%, respectively, with a statistically significant decrease (*p* < 0.05) observed in the 0.2% BAC and 0.5% BAC (*p* < 0.01) groups. In addition, there was statistically significant decrease (*p* < 0.05) in the 0.1% and 0.2% BAC groups compared with the 0.5% BAC group.

### 3.2. Transmission Electron Microscopy

**Figure 5:**
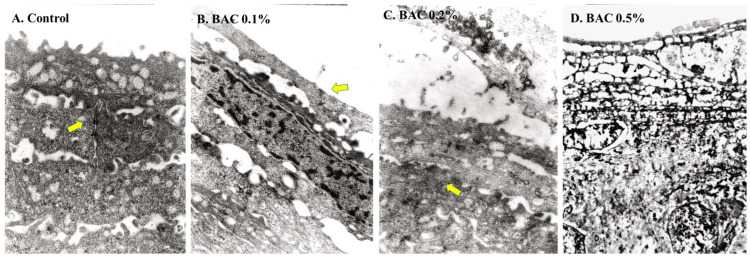
TEM images showing the superficial mouse corneal epithelium with microvilli and junctional complex in the second layer (arrow) (control, (**A**)). In (**B**) (BAC 0.1%), the microvilli seem to disappear, but a tight junction between the superficial cells exists (arrow). The two superficial layers of the epithelium disintegrate, and the tight junctions between the subsequent layers become loose (arrow in (**C**); BAC 0.2%). The superficial layers of the corneal epithelium appear as ghost cells with the total disappearance of microvilli (**D**) (BAC 0.5%). ((**A**–**C**) magnification = 12,000×; (**D**) magnification = 4000×).

TEM examinations of the control eyes showed tightly packed superficial cells with multiple microvilli with the presence of a junctional complex between adjacent cells (Figure 5A). The microvilli decreased from the most superficial cells in the 0.1% BAC group, but not in subsequent layers with the presence of tight junctions between the adjacent superficial cells (Figure 5B). Figure 5C shows a decreased number of epithelial microvilli and the disintegration of two layers of superficial cells with a loss of epithelial tight junctions between the cells. In the 0.5% BAC group, the superficial cells of the epithelium appeared as ghost cells with the shedding of most superficial cells (Figure 5D).

### 3.3. ZO-1 Immunohistochemical Staining

**Figure 6:**
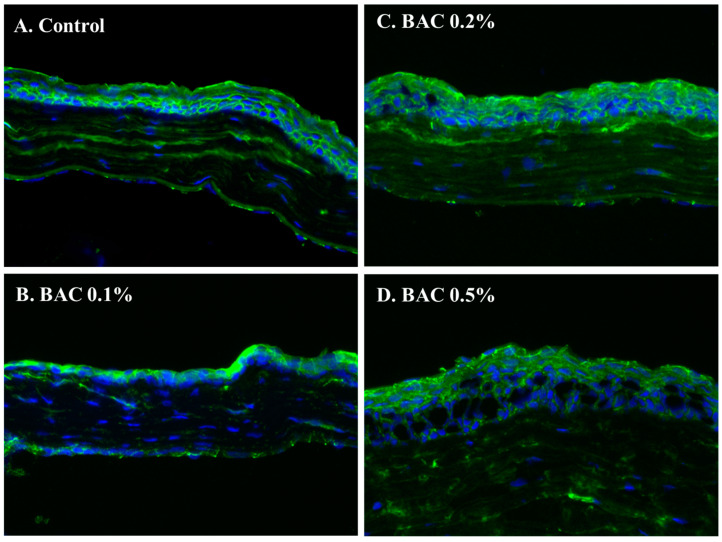
The IHC examination showed uniform intensity in a ZO-1 staining pattern along the perimeter of the cell membranes of the corneal epithelium of HBSS (**A**) and 0.1% BAC (**B**) groups in comparison to the lower intensity and multiple breaks in the 0.2% BAC (**C**) and 0.5% BAC (**D**) groups.

An IHC examination revealed that the ZO-1 staining pattern along the perimeter of the cell membranes in the corneal epithelium was diminished and exhibited multiple breaks in the 0.2% BAC and 0.5% BAC groups when compared to both the control group and the 0.1% BAC group (Figure 6).

## 4. Discussion

In our previous publications, we described and explained the importance of TER to evaluate the corneal irritation and permeability induced by ophthalmic drugs. TER is also used to study the integrity of tissues and cell sheets, such as the intestinal epithelium and Madine–Darby canine kidney cells [19,20].

Most ophthalmic drugs contain adjuvants such as surfactants and preservatives. They are often essential for ocular liquid formulations, solubilizing drugs, and preventing microbial contamination. Some of these adjuvants act as ocular-penetrating enhancers, promoting drug penetration through the strong corneal barrier and modifying the physiochemical property of drugs. At the same time, however, they damage the corneal epithelial structure, especially the transcellular integration of superficial cells, which is mainly maintained by tight junctions. Therefore, the investigation of the effect of ophthalmic drugs and adjuvants on the cornea is important [10,11,12,13].

Our previous innovative methods for measuring TER in rabbits and humans [10,11,12,13] are not applicable for mice because of the small size of the cornea.

While using mice as a model has an obvious disadvantage (their corneal diameter is much smaller than that of humans), the mouse cornea still contains five distinct layers: the epithelium, anterior limiting lamina, stroma, posterior limiting lamina, and endothelium [21]. Although the presence of an anterior limiting lamina has been debated, these layers provide valuable insights for research [22]. Mice are incredibly successful mammals and serve various roles, including as pets, research subjects, vermin, and vectors. Their widespread use in experimental laboratories stems from their genetic homology with humans, small size, and rapid reproductive cycle [23].

The mouse serves as the predominant model organism in human disease research [24]. Researchers have extensively utilized mouse models to gain insights into the underlying mechanisms of various diseases, evaluate the effectiveness of potential drugs, investigate immunological mechanisms, and predict patient responses [25,26].

To the best of our knowledge, this study is the first to innovate TER in the examination and evaluation of murine corneas.

In our previous studies, we proved that the TER of the corneas of both rabbits and humans was statistically significantly decreased within seconds of exposure to the 0.02% BAC concentration [10,11,12,13,14]. In our pilot studies, in preparation for this study), we noticed that the TER of the mouse corneas did not decrease significantly, even at a 0.1% BAC concentration and prolonged for 3 min. In this study, we confirmed that only the 0.2% BAC and 0.5% BAC groups caused a statistically significant decrease in TER after 3 min.

The adherens junctions, desmosomes, and tight junctions of the corneal epithelium form a unique structural and physiological barrier to the external environment [27]. Tight junctions play various essential roles, including regulating the movement of ions, water, and molecules across intercellular junctions. Additionally, they create a physical seal within the epithelial sheet and contribute to maintaining cell polarity [28]. The tight junction complex consists of various proteins, including zonula occludens (ZO), occludin, and claudin. Zonula occludens-1 (ZO-1) is a tight-junction-associated protein that interacts with occludin, forming part of the intricate network of tight junction proteins [29,30]. ZO-1 is expressed in superficial and subsuperficial cell layers of the corneal epithelium and contributes to the barrier function of this epithelium [31,32,33].

The stratified arrangement of the murine corneal epithelium aligns with descriptions found in other mammalian corneas, including those of rats, rabbits, cats, and humans [15,34]. In comparison to the human epithelium, the mouse epithelium contains approximately twice the number of cell layers, primarily due to an increased count of squamous cells [35,36]. The murine corneal epithelium has an average of 13 cell layers, with a thinning of approximately three layers toward the peripheral cornea. Additionally, other epithelial features—such as desmosomal junctions, hemidesmosomes, and the basement membrane—are present in a manner similar to those observed in primates [36].

This increase in the number of squamous cells and the presence of tight junctions in multiple superficial layers may be the cause of resistance of the corneal epithelium of the mice to higher concentrations of BAC in comparison to rabbits or humans. This is confirmed with ultrastructural and immunohistochemical examinations and results.

Based on the above observations, we recommend using rabbits as an ideal animal model for experimental TER measurements instead of mice due to the similarity of their corneal epithelium with that of humans.

While it may seem obvious, selecting an appropriate model is crucial for addressing specific research questions. An ideal disease model faithfully replicates the human condition, encompassing genetic, experimental, and physiological aspects [25].

Establishing stringent standards for conducting and reporting mouse studies is crucial for enhancing reproducibility. Validating the chosen model, implementing proper controls, and meticulously addressing experimental design and statistical analysis are essential steps for maximizing the translational impact of animal experiments. Authors, editors, and reviewers share the responsibility of avoiding the common pitfalls associated with experimental animal use and ensuring that experiments adhere to the highest ethical and regulatory standards [37].

While the debate about the usage of model organisms persists, it remains essential to recognize that these models are simply not human models. However, the insights gained from studies involving model organisms have significantly influenced human health and will undoubtedly continue to do so [25].

## 5. Conclusions

In this study, we succeeded in developing a novel technique for corneal TER measurement in mice, but we think it is not an ideal model for human TER measurements because mouse corneas are sensitive to higher concentrations of BAC compared to human and rabbit corneas. The duplication of the corneal epithelium layers and the presence of tight junctions in more than one layer make the murine corneal epithelium more resistant to external stimuli than human or rabbit epithelia.

## Data Availability

The datasets used and/or analyzed during the current study are available from the corresponding author on reasonable request.
